# High seroprevalence for SARS-CoV-2 among household members of essential workers detected using a dried blood spot assay

**DOI:** 10.1371/journal.pone.0237833

**Published:** 2020-08-14

**Authors:** Thomas W. McDade, Elizabeth M. McNally, Aaron S. Zelikovich, Richard D’Aquila, Brian Mustanski, Aaron Miller, Lauren A. Vaught, Nina L. Reiser, Elena Bogdanovic, Katherine S. Fallon, Alexis R. Demonbreun

**Affiliations:** 1 Department of Anthropology and Institute for Policy Research, Northwestern University, Evanston, Illinois, United States of America; 2 Canadian Institute for Advanced Research, Child and Brain Development Program, Toronto, Canada; 3 Center for Genetic Medicine, Northwestern University, Evanston, Illinois, United States of America; 4 Division of Cardiology, Department of Medicine, Northwestern University Feinberg School of Medicine, Chicago, Illinois, United States of America; 5 Department of Biochemistry and Molecular Genetics, Northwestern University, Evanston, Illinois, United States of America; 6 Division of Infectious Diseases, Department of Medicine, Northwestern University Feinberg School of Medicine, Chicago, Illinois, United States of America; 7 Institute for Sexual and Gender Minority Health and Wellbeing and Department of Medical Social Sciences, Northwestern University, Chicago, Illinois, United States of America; 8 Department of Pharmacology, Northwestern University Feinberg School of Medicine, Chicago, Illinois, United States of America; Centers for Disease Control and Prevention, UNITED STATES

## Abstract

**Objective:**

Serological testing is needed to investigate the extent of transmission of SARS-CoV-2 from front-line essential workers to their household members. However, the requirement for serum/plasma limits serological testing to clinical settings where it is feasible to collect and process venous blood. To address this problem we developed a serological test for SARS-CoV-2 IgG antibodies that requires only a single drop of finger stick capillary whole blood, collected in the home and dried on filter paper (dried blood spot, DBS). We describe assay performance and demonstrate its utility for remote sampling with results from a community-based study.

**Methods:**

An ELISA to the receptor binding domain of the SARS-CoV-2 spike protein was optimized to quantify IgG antibodies in DBS. Samples were self-collected from a community sample of 232 participants enriched with health care workers, including 30 known COVID-19 cases and their household members.

**Results:**

Among 30 individuals sharing a household with a virus-confirmed case of COVID-19, 80% were seropositive. Of 202 community individuals without prior confirmed acute COVID-19 diagnoses, 36% were seropositive. Of documented convalescent COVID-19 cases from the community, 29 of 30 (97%) were seropositive for IgG antibodies to the receptor binding domain.

**Conclusion:**

DBS ELISA provides a minimally-invasive alternative to venous blood collection. Early analysis suggests a high rate of transmission among household members. High rates of seroconversion were also noted following recovery from infection. Serological testing for SARS-CoV-2 IgG antibodies in DBS samples can facilitate seroprevalence assessment in community settings to address epidemiological questions, monitor duration of antibody responses, and assess if antibodies against the spike protein correlate with protection from reinfection.

## Introduction

Serological testing for SARS-CoV-2 IgG antibodies identifies prior viral exposure and, potentially, immunity. Recent surveys suggest a range of seroprevalence rates, with relatively low rates in much of the US [1,2]. Optimal surveillance of seroprevalence ideally avoids contact between community members and health care providers and surveyors, since such contact may carry risk of exposure and discourage survey participation. An alternative to venipuncture blood collection is finger stick dried blood spot (DBS) sampling [3,4]. DBS relies on a finger prick with blood drops captured on filter paper, and DBS can be performed in the home with return of sample by mail. DBS sampling has served as the foundation for nationwide newborn screening programs since the 1960s and is increasingly applied as a minimally-invasive alternative for community health research. The Centers for Disease Control and US Postal Service consider DBS specimens nonregulated, exempt materials for return of samples to laboratories [5].

A robust and quantitative ELISA was granted Emergency Use Authorization from the FDA; this ELISA measures SARS-CoV-2 antibodies in serum and has been determined to not cross-react with other common coronavirus strains [6]. This same ELISA was recently used to detect a higher than expected seroprevalence for antibodies to SARS-CoV-2 among health care workers and patients in a pediatric dialysis unit [7]. We adapted this ELISA to measure IgG antibodies to the receptor binding domain (RBD) of the SARS-CoV-2 spike protein in DBS samples. The RBD is often the target of neutralizing antibodies, although data on the frequency of RBD-binding antibodies with neutralizing activity is still limited [8]. The primary objective of this paper is to describe our protocol and present data on assay performance and validity. In addition, we demonstrate the feasibility and utility of quantifying SARS-CoV-2 antibodies in self-collected DBS with results from a community-based sample enriched with health care workers.

## Methods

A detailed assay protocol is supplied in the S1 File. All research activities were implemented with written informed consent under protocols approved by the institutional review board at Northwestern University (#STU00212457 and #STU00212472).

A community-based sample of 232 adults was recruited through electronic communication initiated by the investigators. Participants completed a brief survey of COVID-19 symptoms and diagnosis, and were provided with materials for DBS collection. DBS sample collection occurred between April 18 and May 20, 2020. All participants returned a blood sample of sufficient quantity and quality for analysis. A set of matched serum and DBS samples was collected from 17 individuals in the study. In addition, DBS samples from 23 individuals presumed to be negative based on the pre-pandemic date of sample collection (2018) were used as negative controls for assay validation. Samples were analyzed in duplicate, with the average result reported.

### Statistical analysis

Statistical analyses were performed with Prism (Graphpad, La Jolla, CA). An unpaired two-tailed t-test was used to compare negative and confirmed positive samples. Error bars represent ± standard error of the mean (SEM). Passing-Bablok regression and bivariate correlation were used to evaluate patterns of statistical association across matched DBS and serum samples.

## Results

**Fig 1:**
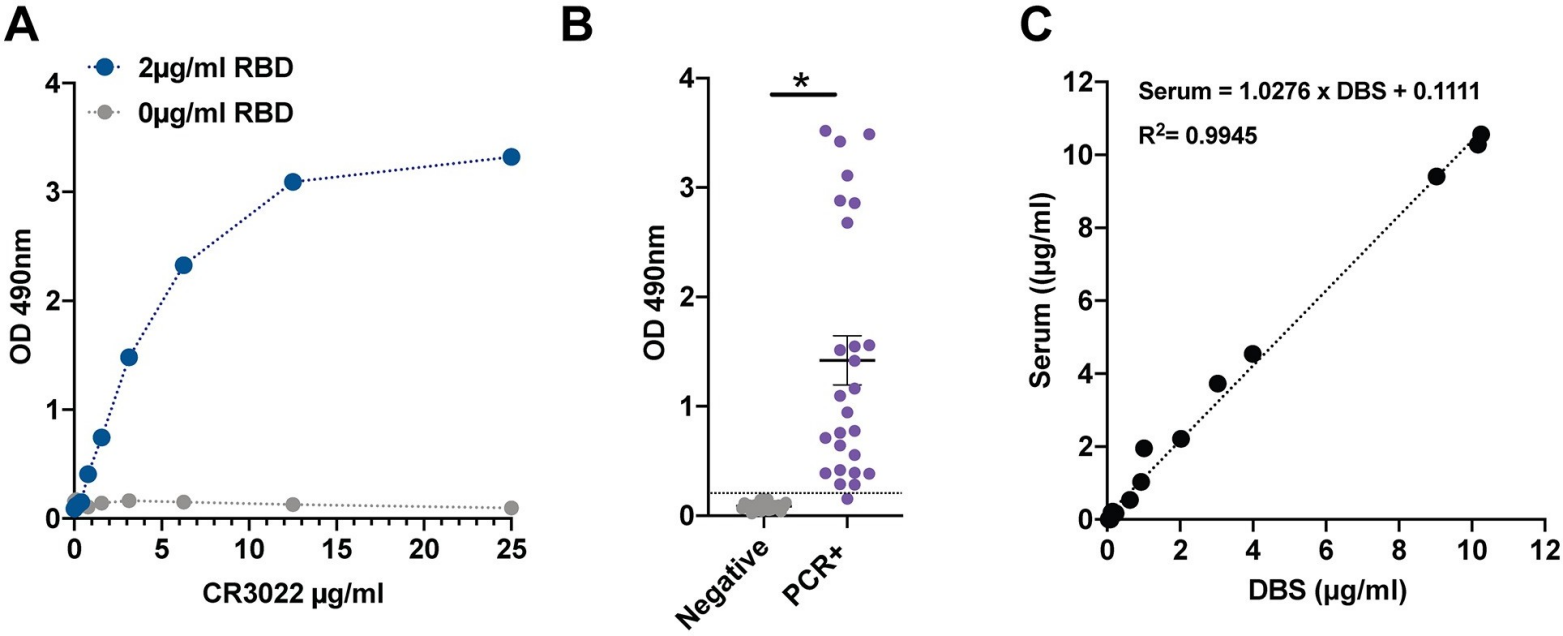
Dried blood spot (DBS) ELISA for the receptor binding protein domain of the SARS-CoV-2 spike protein. **A)** The CR3022 antibody has known reactivity to the receptor binding domain protein. Measurements from DBS samples to which known concentrations of CR3022 antibody were added. **B)** Increased signal readily detected in DBS from virus-positive cases. **C)** Near perfect correlation between DBS and serum IgG levels. *p<0.001.

CR3022, an antibody with defined affinity to the RBD, was used to validate DBS samples in an ELISA. We observed a strong dose-response between CR3022 concentration and optical density (OD) in wells coated with 2.0 μg/mL RBD (Fig 1A), with no response in the absence of RBD antigen. Responses were approximately linear up to 6.25 μg/mL, with a flattening of response at higher concentrations of CR3022. Based on a calibration curve generated from the CR3022 dilution series, the lower limit of detection (LLD) was determined to be 0.032 μg/mL. The cut-off for low seropositivity was set at the OD value produced by the 0.39 μg/ml CR3022 calibrator; this low seropositive cut-off is well above LLD and greater than 3 standard deviations above OD values for known negative samples. The cut-off for full seropositivity was set at the OD value corresponding to the 0.60 μg/ml CR3022 calibrator. The low seropositive cut-off range contained 2 of 30 (6.6%) known PCR+ samples. 90% of the known PCR confirmed positive cases were above the 0.60 μg/ml seropositive threshold.

There was clear distinction between positive and negative samples (Fig 1B). With one exception, all PCR confirmed positive samples were more than 3 SD from the mean OD for the negative samples. Intra-assay variability (percent coefficient of variance (%CV), based on analysis of 10 replicates) at the seropositivity threshold of 0.60 μg/mL was 4.23, and 6.56 and 6.20%CV at 1.56 and 6.25 μg/mL, respectively. Results from matched DBS and serum samples showed tight agreement (Fig 1C).

**Fig 2:**
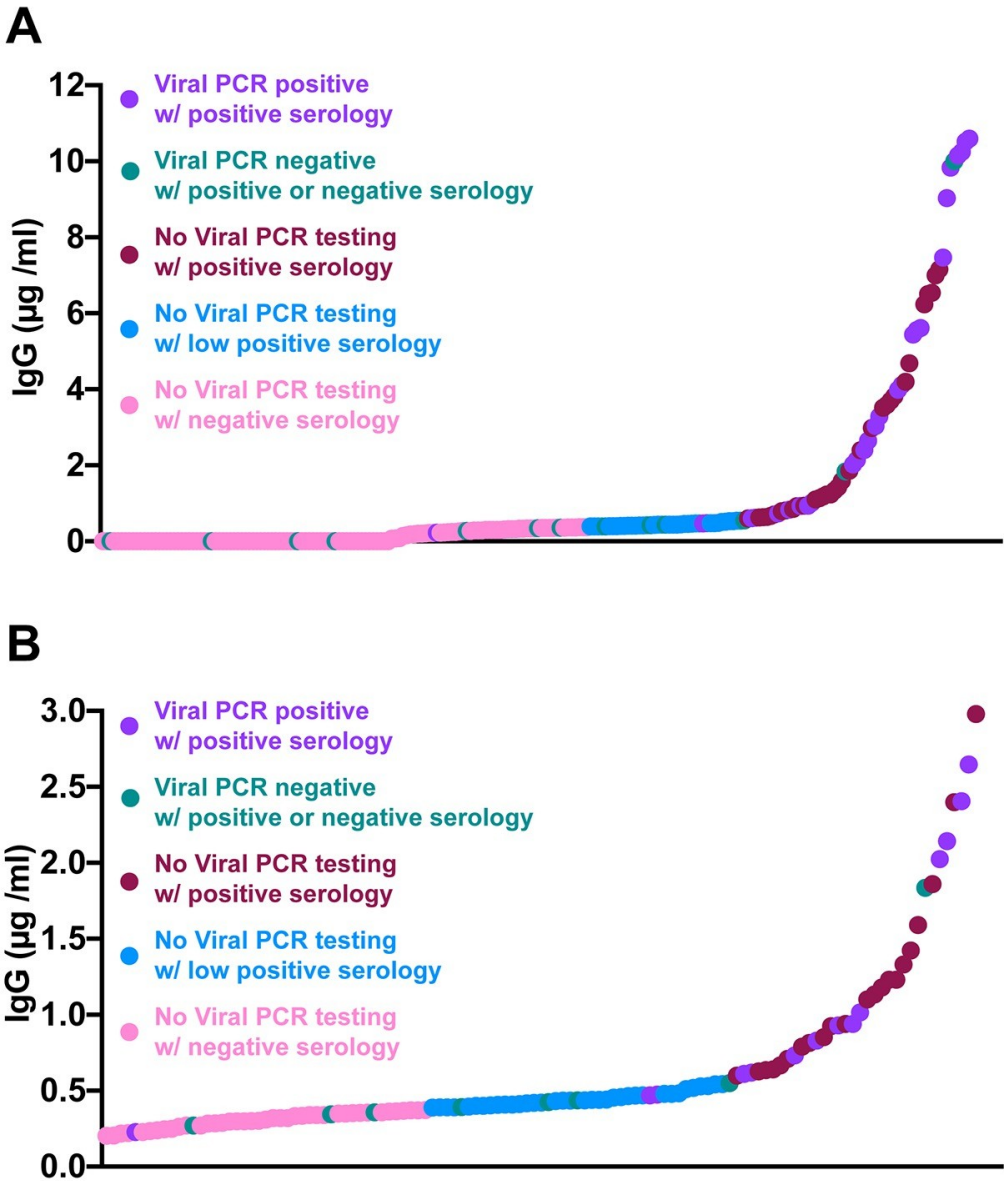
Results from community collected DBS from April—May 2020. **A)** The range of IgG seropositivity detected in DBS samples collected from 30 known virally infected cases (median 28 days after viral test, range 16–43 days) and 202 without documented COVID-19 infection. **B)** Depicts the lower range of DBS detected seropositivity with those OD greater than 0.6 μg/ml considered positive, and less than 0.39 μg/ml considered negative. The range in between was considered low seropositive.

We evaluated IgG levels to RBD protein in 232 DBS samples acquired from the community (Table 1). Thirty cases had previous positive viral PCR testing for COVID-19, with 60–85% reporting chills, fatigue, cough, or headache as the most common symptoms. Twenty-seven of 30 were seropositive with IgG concentrations from 0.6 μg/ml to 10 μg/ml (Fig 2). The median time between serological testing and viral PCR testing was 28 days (range 16–43 days). Two participants had low seropositivity (IgG<0.6 μg/ml but >0.39 μg/ml) and both had asymptomatic infections. The single seronegative participant was pregnant, and 52 days after the first viral PCR test she tested positive for virus again.

**Table 1:** Characteristics of study participants.

	Confirmed positive	Negative/unconfirmed
Number participants	30	202
Mean age (yrs)	36	37
Age range (yrs)	24–74	18–70
Female	26	101
Male	4	101
Viral PCR test positive	30	0
Viral PCR test negative	0	15
No viral PCR testing	0	187
Mean #days post PCR	28	NA
Range #days post PCR	16–43	NA

Distribution of participants in a community-based sample, and descriptive statistics for participants with a confirmed PCR positive case of COVID-19 and without a confirmed case of COVID-19 (negative/unconfirmed).

Among the remaining 202 participants, none of whom tested positive for SARS-CoV-2 with PCR, 60% reported some COVID-19-like symptoms with headache, fatigue, or rhinorrhea being most common. Fifteen of these participants were PCR tested and determined viral negative; of these, 9 were determined to be seronegative, 4 were low seropositive, and 2 were seropositive in DBS. Only one participant required hospitalization for dyspnea (0.8%), and this individual tested viral negative and was low seropositive.

**Fig 3:**
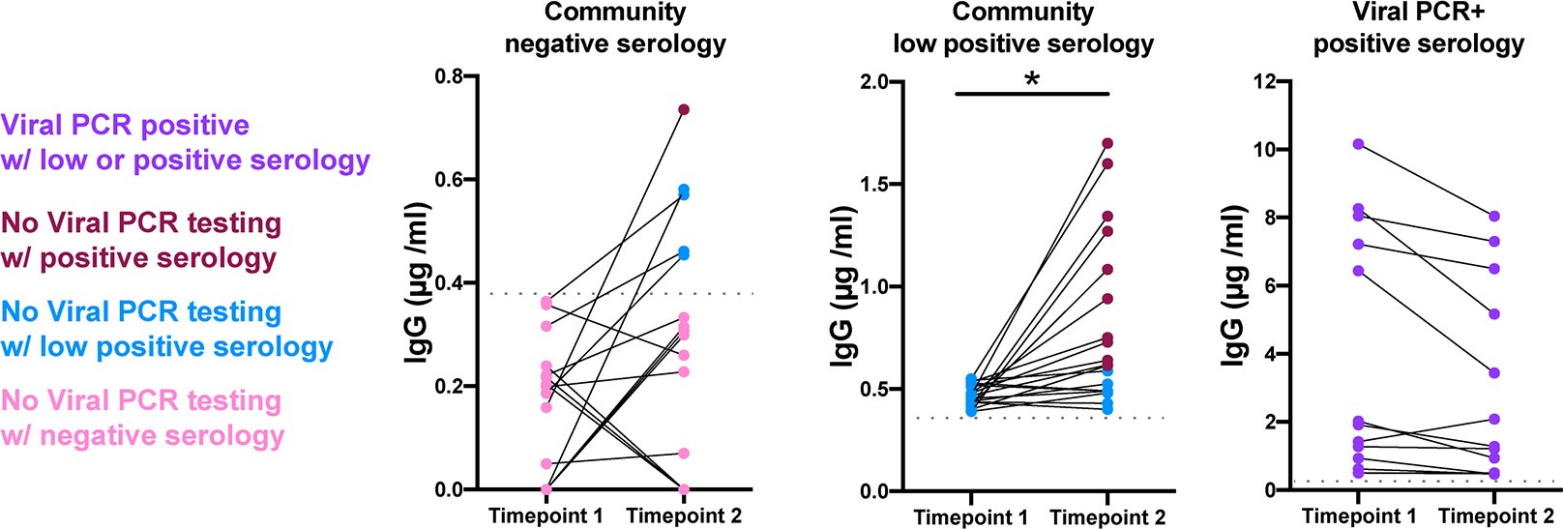
Seroconversion documented in repeat DBS sampling in non-confirmed SARS-CoV-2 and PCR positive participants. Increased IgG concentrations in 10/28 (35%) seronegative samples, 5 of which became low positive or seropositive (18%) upon resampling (median 14 days; range14-23 days). After 2–3 weeks (median 14 days; range 13–23 days), 15/19 (79%) low seropositive participants had increased IgG concentrations with 11/19 (58%) becoming seropositive. 13 of 13 (100%) of known SARS-CoV-2 viral PCR+ participants remained low seropositive or seropositive upon resampling (range 14–21 days; median 47 days post positive viral swab test). Dotted grey line marks the low positive cut-off value. * p < 0.05.

Follow-up DBS samples were acquired from 28 seronegative and 19 low seropositive participants, approximately 2–3 weeks (range from 13–23 days) after the original DBS sampling date from May 5 through June 4, 2020. IgG to RBD increased in 10 (35%) of the seronegative participants, shifting 1 to seropositive and 4 to low seropositive (Fig 3). Fifteen of 19 (79%) low seropositive samples increased IgG, with 11 of 19 (58%) becoming seropositive. Thirteen of 13 (100%) known viral positive SARS-CoV-2 participants remained low seropositive or seropositive upon resampling (mean 41 days post positive viral swab test).

**Fig 4:**
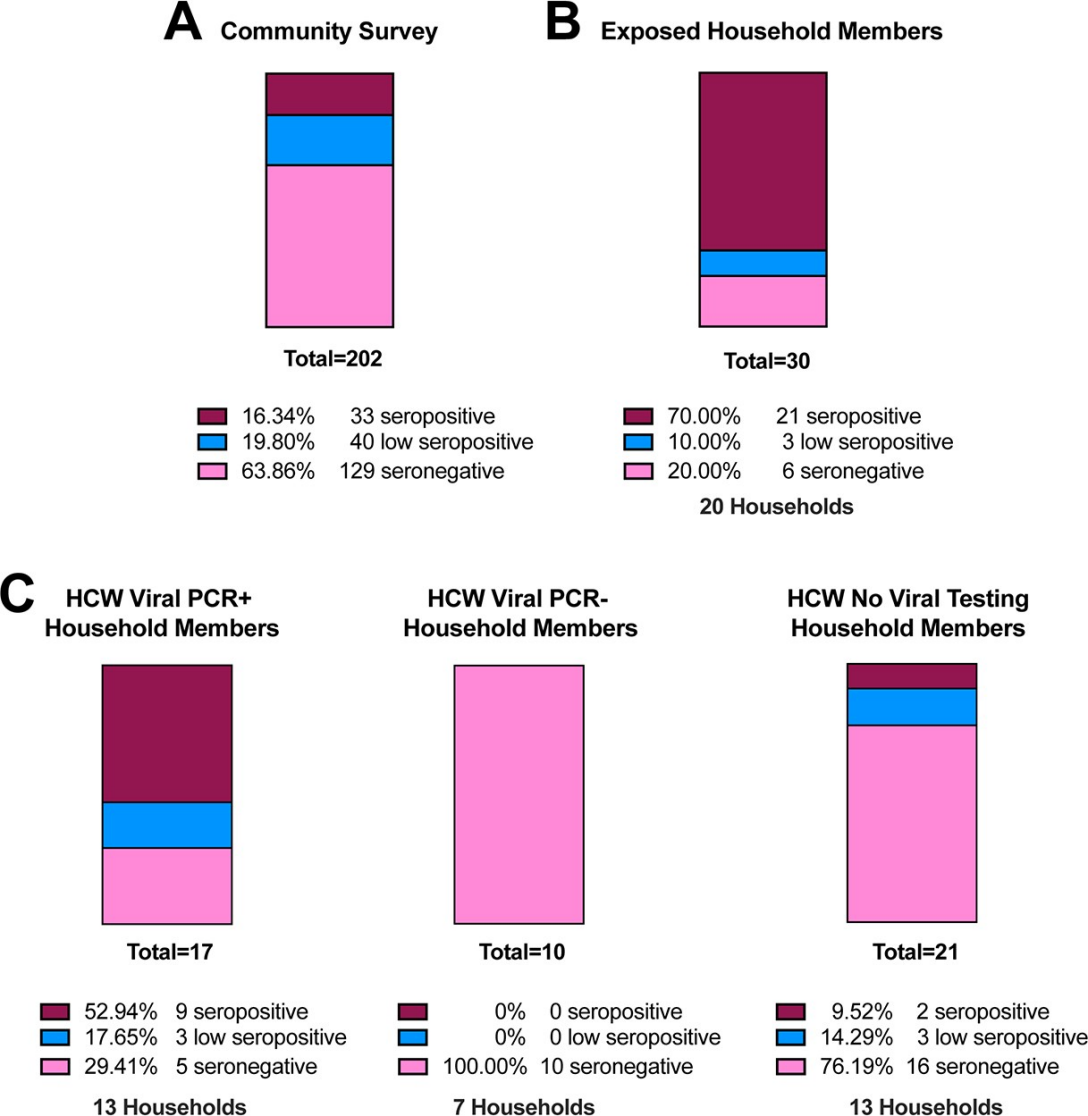
High seroconversion rates in household members of index COVID-19 cases. **A)** Seroprevalence in 202 samples collected from the community, which includes health care workers and first responders, none of which were confirmed SARS-CoV-2 viral positive. **B)** Of 30 COVID-19 exposed household members, 70% were seropositive and 10% low seropositive. **C)** Increased seroconversion in household members of known viral PCR positive healthcare workers (53% seropositive and 18% low positive) compared to 100% seronegative in known viral PCR negative healthcare worker households. Household members of healthcare workers with no viral testing had exposure rates more similar to the community acquired rates.

For the 202 unconfirmed/negative individuals, IgG concentrations ranged from 0–10 μg/ml, with 33 (16%) seropositive, 40 (20%) low seropositive, and 129 (64%) seronegative (Fig 4A). In the sample 30 participants shared households with individuals confirmed to have SARS-CoV-2 through a viral PCR test. The virus positive individuals in these households were disproportionately healthcare workers or first responders (19/20). Among the 30 household members, 21 (70%) were seropositive, 3 (10%) were low seropositive, and 6 (20%) were seronegative (Fig 4B).

A number of participants were from a single healthcare facility in which a number of healthcare workers tested positive for virus while others were found to be viral negative between March 24 to April 3, 2020. Those healthcare workers that remained asymptomatic were not tested for active virus. We evaluated the seroconversion of 48 household members from 33 essential healthcare worker households, 40–50 days post the initial exposure date (Fig 4C). Participants sharing a household with a known COVID-19 positive healthcare worker had a high seroconversion rate with 9/17 (53%) seropositive, 3/17 (18%) low seropositive, and 5/17 (29%) seronegative. This was in contrast to 10/10 (100%) of household members remaining seronegative when sharing a household with a virus-negative health care worker. Household members that resided with healthcare workers that were not tested for virus were 2/21 (10%) seropositive, 3/21 (14%) low seropositive, and 16/21 (76%) seronegative. These data show a high transmission rate among households of front-line essential workers and the dynamic nature of seroconversion in the community.

## Discussion

COVID-19 is now widely acknowledged to have high rates of community spread. Strategic testing in community-based settings is critical for tracking spread of SARS-CoV-2 and for identifying the factors that mitigate transmission. We have validated a DBS assay to facilitate large-scale serological testing of SARS-CoV-2 IgG antibodies, and results from our feasibility study document a high rate of household transmission. In addition, based on Hains et al. [7] and the data here, indeterminate levels of IgG merit retesting and current estimates of seroprevalence may underestimate community spread.

Because of their potential risks to patients, health care workers and first responders generally had priority for viral PCR testing to ascertain acute infection. However, limited supplies of viral testing materials often restricted access to viral PCR testing during March and April 2020, which likely resulted in under-testing and under-reporting of COVID-19 cases. Serological testing is positioned better to inform viral exposure especially during the interval when viral PCR testing was less available or weeks after symptom onset. Furthermore, improved and reliable serological surveys, combined with close clinical follow up, can elucidate whether serological status predicts immune protection from repeat infection or disease. DBS sampling can aid in these surveys.

Advantages of DBS include a low cost and non-invasive approach to blood collection. DBS samples do not require special handling prior to transport and analysis, and IgG antibodies remain stable in DBS for at least 8 weeks at room temperature [3,9]. While point-of-care lateral flow immunoassay tests share some of the advantages of DBS sampling, they are difficult to implement in the home and they do not provide the same level of accuracy and quantification as lab-based tests for SARS-CoV-2 antibodies [10]. Limitations of DBS include small sample volume, and the possibility for errors in self-collection (i.e., blotting, smearing of blood on filter paper) that can interfere with quantification.

Widespread serological testing is needed to more accurately assess viral spread. While our sample is not representative of the general population, the observation of high household seroconversion, using an assay restricted to a small component of the viral spike protein, suggests there may be higher exposure to COVID-19 among some members of the community than previously appreciated.

## Supporting information

S1 Data (XLSX)

S1 File (DOCX)
