# Supplementary material for: High seroprevalence for SARS-CoV-2 among household members of essential workers detected using a dried blood spot assay
Source: PLoS One. 2020 Aug 14;15(8):e0237833. doi: 10.1371/journal.pone.0237833 (PMC7428174; doi:10.1371/journal.pone.0237833)
Supplement: S1 File — (DOCX) [file pone.0237833.s002.docx]

**Supporting information**

**Detailed Methods**

DBS samples are eluted overnight and transferred to microtiter wells, which were previously coated with SARS-CoV-2 RBD antigen. Goat anti-human IgG-HRP binds to the RBD-antibody complexes in each well, and color forms with the addition of chromogenic substrate. The absorbance of the solution is read at 490nm.

***Reagents and supplies***

*Filter papers*  Whatman #903 (Cytiva #10534612). These cards are approved by the FDA as Class II medical devices for diagnostic applications, and the CDC implements an independent quality control analysis of each card lot to confirm thickness, flow-rate, absorbency, and purity.

*Hole punch* A semi-automatic pneumatic system with 5mm punch (Analytical Sales and Services #327500, Flanders, NJ) was used. Manual punches are also readily available that produce 3/16 inch discs (4.8mm) (e.g., McGill #MCG53600C). A 5 mm punch will contain approximately 5uL of serum [1, 2].

*Microtiter plates* DBS samples are eluted in Corning 96 Well TC-Treated Microplates (Sigma #CLS3599). For coating with antigen: Immulon 4 HBX polystyrene 96-well flat bottomed, high-binding plates (ThermoFisher Scientific #3855).

*Coating antigen* The plasmid for the receptor binding domain (RBD) of the spike (S) glycoprotein gene from SARS-CoV-2, Wuhan-Hu-1 (GenBank: MN908947) was generously provided by Dr. Florian Krammer (Mt. Sinai Medical School, NY) and is described previously.[6] The construct was made by fusing the N-terminal S protein signal sequence to the spike RBD (amino acids 419 to 541) with a C-terminal hexa-histidine tag. The sequence was codon optimized and subcloned into the pCAGGS mammalian expression vector. Recombinant RBD protein was generated by Evotec using the Expi293F cell line and standard methods (Princeton, New Jersey). Briefly, EXPI293 cells were transfected with purified, endotoxin free plasmid DNA. Four L of media was purified using IMAC chromatography 72 hours post transfection. The final recovery of purified recombinant RBD protein was ~195mg in PBS with an endotoxin level at ~ 1EU/mg, with a purity >95%.

*Detection antibody* Anti-Human IgG (Fab specific)-Peroxidase antibody produced in goat (Sigma-Aldrich #A0293).

*Positive control* IgG antibody to SARS-CoV S protein (CR3022 antibody, Creative Biolabs #MRO-1214LC).

*Coating buffer* Phosphate buffered saline (1x PBS; Fisher BP24384 or equivalent)

*Wash buffer* PBS, 0.1% Tween 20 (PBS-T; Fisher BP337-500).

*Blocking solution* 0.1% PBS-T, 3% milk (w/v; American Bio AB10109-01000).

*Chromogenic substrate* SIGMA*FAST™* OPD (Sigma-Aldrich #P9187). One silver and one gold tablet per 2 plates in 20 mL dH2O.

*Stop solution* 3.0M Hydrochloric acid (HCl).

*Antibody diluent*  0.1% PBS-T, 1% milk (w/v).

***Preparation of materials***

DBS calibrators are prepared as follows: 1) Dilute CR3022 antibody to desired concentrations in PBS; 2) Collect EDTA whole blood from a negative donor; 3) Add CR3022 dilutions to aliquots of whole blood, mixing gently to avoid hemolysis; and 4) Transfer with a pipet (65 μL/drop) to labeled #903 cards. Dry overnight at room temperature and store at -23°C. It is important to minimize the volume of CR3022 dilution that is added to whole blood, as large volumes (>5% of total volume) will lower hematocrit and adversely affect how the sample is absorbed on the filter paper.

***Protocol***

The 96-well plate is coated with antigen as follows: Dilute RBD to 2 µg/mL in PBS, add 100 µL to each well, seal, and incubate overnight at 4°C. DBS samples are eluted overnight. Punch out one 5.0 mm disc of each DBS calibrator, control, and sample, and place in elution plate. Add 250 µL PBS, seal the plate, and elute overnight at 4°C.

The following day, remove plate and wash 4x with 300 µL PBS-T (BioTek ELx50 or equivalent). Add 200 µL per well 0.1% PBS-T, 3% milk. Block covered at room temperature for two hours. Aspirate.

At one hour, remove DBS samples from refrigerator and rotate at 300 rpm (Heidolph Titramax 101 or equivalent). After removing blocking solution, transfer 100 µL eluate from DBS calibrators, controls, and samples to the coated 96-well plate. Cover the plate and incubate at room temperature for two hours. Wash the plate four times with PBS-T.

Prepare a dilution (1:3000) of detection antibody in 0.1% PBS-T, 1% milk. Add 100 µL working antibody solution to each well. Cover and incubate at room temperature for 60 minutes. Wash four times as before.

Prepare chromogenic substrate by dissolving one set of tablets in 20mL dH2O. Do not add silver tablet until ready to use. Add 100 µL chromogenic substrate to each well. Cover the plate, protect from the light, and incubate for 10 minutes at room temperature. Add 50 µL 3M HCl stop solution to each well. Incubate 5 minutes at room temperature. Read the absorbance (optical density, OD) at 490 nm (BioTek ELx808 or equivalent). OD > 0.60 µg/ml CR3022 is considered positive, less than 0.39 µg/ml CR3022 is considered negative, and values between are considered low seropositive. Sample IgG concentration (µg/ml) is calculated from the linear regression of the CR3022 calibration curve.

**References**

1. Adam BW, Alexander JR, Smith SJ, Chace DH, Loeber JG, Elvers L, et al. Recoveries of phenylalanine from two sets of dried-blood-spot reference materials: prediction from hematocrit, spot volume, and paper matrix. *Clinical Chemistry*. 2000;46:126-8.

2. Mei JV, Alexander JR, Adam BW, Hannon WH. Use of filter paper for the collection and analysis of human whole blood specimens. *Journal of Nutrition*. 2001;131:1631S-6S.
